# Pituitary Apoplexy: a re-appraisal of risk factors and best management strategies in the COVID-19 era

**DOI:** 10.1007/s11102-024-01420-0

**Published:** 2024-08-05

**Authors:** Andre E. Boyke, Michelot Michel, Adam N. Mamelak

**Affiliations:** 1 Department of Neurosurgery, Cedars-Sinai Medical Center, 127 S San Vicente Blvd, Los Angeles, CA A6600 USA; 2 University of Florida College of Medicine, Gainesville, FL USA

**Keywords:** Pituitary apoplexy, Transsphenoidal surgery, Medical management, Outcomes, Review

## Abstract

Pituitary apoplexy (PA) is a clinical syndrome caused by acute hemorrhage and/or infarction of the pituitary gland, most commonly in the setting of a pituitary macroadenoma. PA generally presents with severe headache, nausea, vomiting, visual disturbance, and, in more severe cases, altered mental status. Many factors have been attributed to the risk of developing PA, including most recently, numerous reports showcasing an association with COVID-19 infection or vaccination. Initial management of PA includes evaluation and correction of deficient hormones and electrolytes and an assessment if surgical decompression to relieve pressure on optic nerves and other brain structures is needed. While prompt recognition and treatment are crucial to avoid morbidity and mortality, in the modern era, PA is less commonly considered a true neurosurgical emergency requiring immediate (< 24 h) surgical decompression. Traditionally, surgical decompression has been the standard of care for significant mass effects. However, several studies have shown similar outcomes in visual and hormonal recovery with either surgical decompression or conservative medical management. Unfortunately, most evidence on optimal management strategies is limited to retrospective case series, small prospective studies, and one multi-center observational study. This review aims to provide the most up-to-date evidence on the role of COVID-19 in PA and best management strategies.

## Introduction and historical overview

Pituitary apoplexy (PA) is a clinical syndrome caused by acute hemorrhage and/or infarction of a tumor in the pituitary fossa. The first formal use of the term PA was adopted by Brougham et al. in 1950 when authors used it to reference a case of necrosis and hemorrhage that occurred in patients with pituitary tumors [1]. Since then, there have been numerous reports and case series describing the clinical presentation, treatment, and outcome of PA patients. PA, although rare, has traditionally been considered a life-threatening condition that requires urgent medical and surgical intervention to prevent morbidity and mortality. However, with improved early recognition, understanding of the underlying disease process, and effective therapies, the treatment strategies for PA have evolved considerably. Consequently, there is considerable debate in the literature regarding the management of acute PA, with discussions often centered on the merits of medical versus surgical interventions. This review will provide a comprehensive assessment of PA, outlining the most evidence-based approach to managing this condition.

## Epidemiology

PA is most common in the 5th decade of life [2–4], with most patients between 37 and 58 years old [3]. Some studies suggest that PA is more common in men, although female predominance has also been observed in others [5, 6]. Prior reports have highlighted varying incidence rates. Among patients with known pituitary adenomas, the rate of PA ranges from 1.5 to nearly 28% [5, 7, 8]. This wide range has been attributed to the consideration of symptomatic versus subclinical or non-symptomatic PA cases, with the latter being diagnosed following neuroimaging studies and is more frequent [7]. For purposes of this review, we consider PA an acute clinical syndrome with clear symptoms related to acute hemorrhagic and/or infection and do not consider asymptomatic bleeding found on routine imaging as part of this clinical spectrum.

## Pathophysiology

Most PA occurs in previously undetected or clinically asymptomatic non-functioning pituitary macroadenomas [9]. After non-functioning adenomas, prolactinomas, and growth hormone-secreting tumors comprise the second and third most common pituitary neoplasms seen in the setting of PA (Table 1) [10–12]. Macroadenomas have fragile, relatively immature blood vessels [13, 14]. Turner et al. compared the expression of CD31, an endothelial cell marker, revealing that pituitary adenomas typically exhibit lower vascular density than normal, reducing bleeding risk, although invasive macroprolactinomas demonstrate increased vascularity, which could influence observed bleeding risks [14]. 

**Table 1:** Hypersecreting adenomas reported amongst PA cases in select studies

Author	Year	No. of PA cases	Cohort	Hypersecreting Adenomas, (%)		
				Prolactin	Growth hormone	Cortisol
Randeva et al. [15]	1999	35	All	5.7	8.6	5.7
Sibal et al. [16]	2004	45	All	4.4	2.2	6.7
Grzywotz et al. [2]	2017	60	All	10	6.7	6.7
Rutkowski et al. [17]	2017	32	All	9.4	3.1	0
Marx et al. [18]	2021	19	Surgery	15.8	0	0
		27	Conservative	33	3.7	3.7
Shepard et al. [19]	2021	64	All	6.3	0	0
Mamelak et al. [4]	2024	67	Surgery	4.5	0	1.5
		30	Conservative	13	3.3	0

Sudden and rapid bleeding in a tumor and/or infarction of that same tumor (either from ischemia or mass effect) results in an acute rise in intrasellar pressure. Furthermore, infarction of pituitary tissue may cause swelling on its own. This expanding mass within the bony limits of the sella and skull results in rapid compression of surrounding tissues. Superior extension of an expanding pituitary mass is the primary mechanism leading to visual field loss, most commonly bitemporal hemianopsia resulting from optic chiasm compression. Alternatively, unilateral hemianopsia, asymmetric loss, or even blindness may develop depending on the severity of nerve compression. Expansion laterally may exert a mass effect on the cavernous sinus, leading to diplopia caused by oculomotor palsies of cranial nerves III, IV, or VI, with incidence rates ranging from 25 to 39% [16, 20]. Although less common in the modern era, superior extension can also compress other brain structures, resulting in diminished consciousness, coma, hydrocephalus, and even death [21]. Furthermore, there is a risk of hemorrhage extending into the basal cisterns, potentially leading to vasospasm and meningeal irritation, worsening headaches, and nausea [22]. 

### Precipitating factors

While most cases of PA occur sporadically, several reports, often limited to small retrospective studies and case reports, have postulated potential factors. One of the better-recognized precipitating risk factors for PA is cerebral hypoperfusion or hemodynamic disturbances due to major surgery, most notably cardiovascular procedures [23]. Other medical conditions and medications such as hypertension, hemorrhagic pregnancy, anticoagulation use, and endocrine function tests have been reported as potential risk factors for PA onset [15, 24]. Sphenoid sinus mucosal thickening on MRI scans in patients with PA is now a well-recognized co-occurrence [25, 26]. It is unclear if this mucosal thickening is a precipitating factor or if PA may induce inflammatory changes in the sphenoid mucosa.

There is also a small risk of hemorrhage following subtotal adenoma resection, which can produce symptomatic PA [27–29]. Furthermore, alternative treatments for adenomas, such as radiation therapy, have also been implicated in post-procedure hemorrhage. Fu et al. retrospectively reviewed 751 consecutive pituitary adenoma patients treated with gamma knife radiosurgery and found that 55 (7.3%) patients developed new or worsened pituitary hemorrhage within a median time of 18.9 months [30]. 

### SARS-CoV-2

Several cases of PA in the setting of a confirmed SARS-CoV-2 infection or vaccination have been reported (Table 2). In most cases, the presentation is like that of other cases of PA, with similar co-morbidities and outcomes. Early in the pandemic, we reported a case of PA in pregnant women with COVID-19 [31], raising the possibility that the COVID-19 infection may have contributed to PA in this setting. Since then, several other cases have been reported. Not surprisingly, most patients had a clinically silent pre-existing adenoma, with two reported cases presenting with a hypersecreting prolactinoma [32, 33]. Given the high incidence and prevalence of COVID-19 in the general population, it is unclear if there is a causal relationship between infection and/or vaccination and subsequent development of PA.

**Table 2:** Cases of PA in the setting of confirmed COVID-19 infection or vaccination reported in the literature

Author	Year	Age	Sex	Co-morbidities	PA onset after vaccination	Symptoms	Time to PA onset from infection or vaccine	Treatment	Hormonal Defects	Follow-up
Chan [31]	2020	28	F	Pregnant	N	HA blurry vision	Same	C + S	NR	NR
dos Santos e Santos [34]	2020	47	M	None	N	HA	3 weeks	S	ACTH	NR
Solorio-Pineda [35]	2020	27	M	None	N	HA ↓visual acuity	4 days	C	FSH/LH	Death due to Resp. failure
LaRoy [36]	2021	35	M	None	N	HA	3 days	C	NR	
Martinez-Perez [37]	2021	54	F	None	N	HA, blurry vision	1 week	S	ACTH TSH	1 month: Improved vision
		56	M	HTN	N	HA diplopia	10 days	S	ACTH TSH	6 weeks: Resolution of diplopia
		52	M	HTN	N	HA, bitemporal hemianopsia	NR	S	Pan hypopituitarism	NR
Katti [38]	2021	46	M	None	N	Sudden vision loss	Same	C	NR	NR
Ghosh [39]	2021	44	F	None	N	HA blurry vision	6 days	C	ACTH	
Bordes [40]	2021	65	F	HTN, Fibromyalgia	N	HA nausea/emesis	1 month	C	ACTH TSH	6 months: Stable
Liew [41]	2021	75	F	Irritable bowel syndrome	N	HA	6 weeks	C	Pan hypopituitarism	Y
Kamel [42]	2021	55	M	HTN, DM Pituitary adenoma	N	HA ptosis ↓ vision L eye	6 days	S	HRT*	1 week Death due to COVID pneumonia
Murvelashvili [43]	2021	51	F	None	Y After 2nd vaccination (Moderna)	Nausea/emesis, epigastric pain, ↓ libido	2 days	C	ACTH FSH/LH TSH	1 month: MRI with reduction in PA volume
Balmain [32]	2022	79	M	None	N	HA visual field deficit	8 weeks	C	ACTH TSH	5 months: Hypopituitarism VF defect stable
		69	F	HTN, Pulmonary embolism	N	Abnormal visual fields	16 weeks	C	ACTH TSH	6 months: Stable clinical exam
		64	F	DM	N	HA abnormal visual fields	Same	C	ACTH	Lost to follow up
		63	F	None	N	HA ↓visual acuity	Same	C	ACTH TSH	3 months: Complete visual recovery
		71	F	HTN, HLD, Hypothyroidism	N	HA complete blindness	Same	S	ACTH TSH	2 months: Visual acuity improved
		75	F	HTN, DM	N	HA	12 weeks	C	ACTH	3 months: No HA, hormonal replacement
		47	F	None	N	HA bitemporal hemianopsia	4 weeks	C	ACTH	6 weeks: No HA, hormonal replacement
		72	M	None	N	HA bilateral blindness	7 weeks	S	ACTH	7 months: Partial recovery of vision
Aliberti [44]	2022	50	M	None	Y, following 3rd vaccination (Moderna)	Nausea/emesis	1 day	S	ACTH FSH/LH	4 months: Resolution of symptoms, Full recovery of hormones
Pinar-Gutierrez [45]	2022	37	F	None	Y, following 1st vaccination (ChAdO)	HA	4 days	C	NR	3 weeks: Symptoms resolved
Zainordin [46]	2022	24	F	None	Y, following 2nd vaccination (AstraZeneca)	HA	24 h	C	GH	3 weeks: HA improved
Hazzi [47]	2023	65	M	None	N	HA, CN III, IV, VI paresis	13 days	S	Pan hypopituarism	6 months: Stable, improvement in visual field
		61	F	None	N	Right CN III paresis	6 days	C + S	ACTH TSH	2 months: CN III paresis improved
		89	M	HTN, HLD	N	HA	2 months	C	Pan hypopituitarism	6 months: Reduction in lesion
Roncati [33]	2023	28	F	None	Y, Following 2nd vaccination (AstraZeneca)	HA amenorrhea	24 h	C	↑PRL	3.5 months Improving, Menses resumed

Legend: HA = headache; CN = cranial nerve; Y = yes; N = no; C = conservative management; S = Surgery; F = female; M = Male; DM = diabetes mellitus; HTN = hypertension; HLD = Hyperlipidemia; NR = not reported; ACTH = adrenocorticotrophic hormone (pituitary related cortisol deficiency); TSH = thyroid stimulating hormone (pituitary related thyroid hormone deficiency); PRL = prolactin

There have been several proposed mechanisms by which SARS-CoV-2 infection might induce PA onset. Coronavirus uses ACE-2 receptors to enter cells, including those in the central nervous system (CNS) [48, 49]. This infection might trigger an immune response, causing damage to the gland. Alberti et al. described a case of PA in which SARS-CoV-2 proteins were located next to pituitary blood vessels on immunohistochemical analysis, suggesting some element of antigen cross-reactivity leading to hypophysitis-induced PA [44]. The investigators also observed lymphocytic cell infiltrate. SARS-CoV-2 infection leads to an overall system immune response, which can indirectly lead to PA onset. The inflammation associated with a severe viral infection can activate the hypothalamic-pituitary-adrenal (HPA) axis. This activation could lead to hypophysitis.

The association between COVID-19 infection and coagulation disorders has also been well-documented in the literature. von Willebrand factor (vWF) is upregulated in COVID-19, highlighting the role of endothelial involvement. ADAMTS13 is a metalloprotease that cleaves high-molecular-weight vWF. Downregulation of its expression has been observed in COVID-19. This results in an aberrant ratio of vWF to ADAMTS13, which is fundamental to the pathogenesis of COVID-19-associated coagulopathy [50]. This disturbed ratio contributes to the hypercoagulable state observed in severe COVID-19 cases, which can lead to thrombosis in the blood vessels supplying the pituitary gland, causing infarction and subsequent hemorrhage. Moreover, common comorbidities in COVID-19 and PA cases include hypertension and diabetes mellitus, both linked to endothelial dysfunction [51]. 

Far fewer cases of PA following vaccination for COVID-19 have been reported in the literature, with three cases [33, 45, 46] occurring following a dose of the AstraZeneca (Cambridge, UK) vaccine [33, 45, 46] and two cases following the Moderna (Cambridge, MA, USA) vaccine [43, 44]. These patients had no prior medical history or comorbidities commonly associated with PA. Vaccination for coronavirus may induce thrombosis and bleeding due to Vaccine-Induced Thrombotic Thrombocytopenia syndrome (VITT) [52]. Although an extremely rare syndrome, VITT is most commonly observed following administration of viral-vector-based vaccines [53]. However, it is likely that this phenomenon is more complex, given that Zainordin et al. reported a case of PA following vaccination that presented with a normal coagulation profile [46]. 

Taken as a whole, these limited data suggest that COVID-19 infection and/or vaccination may be a predisposing risk factor for the development of PA and should be added to that list for future investigation. However, at present, no definitive statement can be made regarding the contribution of COVID-19 to the development of PA.

### Clinical assessment and diagnosis

The most commonly reported symptoms of PA, in descending order of frequency, include headache (86%), visual disturbances (62%), nausea/vomiting (40%), and extra-ocular palsies (25%) [54]. Most of these symptoms have a rapid onset, much like aneurysmal subarachnoid hemorrhage. Importantly, the spectrum of PA encompasses severe symptoms such as blindness, coma, and death.

The constellation of these symptoms often leads to differential diagnoses, which include acute subarachnoid hemorrhage (SAH) and meningitis. Cerebrospinal fluid (CSF) analysis may not distinguish between SAH, bacterial meningitis, and pituitary apoplexy (PA). Based on the aforementioned clinical features, prompt evaluation of suspected PA should include a comprehensive hypothalamic-pituitary axis endocrine assessment, visual field and acuity testing, oculomotor examination for cranial nerve deficits, and consciousness evaluation. MRI should be obtained as soon as feasible, though a CT scan, often more readily available, can demonstrate acute sellar hemorrhage.

Computed tomography (CT) is often used to assess neurological anatomy in patients with sudden severe headaches, identifying an intrasellar lesion in 80% of cases. However, magnetic resonance imaging (MRI) remains the preferred imaging study. Within the first 7 days post-PA, lesions typically appear iso- to hypointense on T1-weighted images and hypointense on T2-weighted images. During the subacute phase, from 7 to 21 days, lesions may become hypointense on both T1 and T2 images due to methemoglobin accumulation. Intravenous gadolinium administration can reveal a contrast enhancement around the pituitary gland, known as the “pituitary ring sign.” [55] Beyond 21 days, signal intensities may become more heterogeneous as the gland undergoes blood product degradation and fibrosis.

Assessment of endocrine abnormalities is a crucial step in clinical assessment. Acute cortisol deficiency is a frequent and critical complication observed in patients with PA, occurring in 50–80% of cases [4, 16, 56, 57]. This condition can lead to severe hemodynamic instability and hyponatremia, necessitating prompt identification and management. Immediate administration of glucocorticoid replacement is essential for patients exhibiting signs of PA to avert an adrenal crisis. Beyond cortisol, supplementation of deficient hormones in the relatively early stages of management is of critical importance to avoiding other endocrinopathies such as hyper- or hyponatremia.

To categorize the clinical manifestations of PA and define which groups of patients were most likely to undergo surgery, the United Kingdom Pituitary Apoplexy Guidelines Development Group introduced the Pituitary Apoplexy Score (PAS) [58]. The PAS quantifies severity using consciousness level, visual acuity, field deficits, and ocular paresis. Developed through a retrospective review to standardize treatment, the score remains unvalidated for outcome prediction. In the modern era, most patients present with a PAS of 4 or less, so it is unclear if this scoring impacts advising best treatment approaches.

## Management strategies and outcomes

### Surgical versus conservative management

The debate over the optimal treatment strategy for patients with PA has evolved significantly since the initial view by many that acute neurosurgical intervention was almost always needed [59]. Numerous retrospective and a few prospective studies have tried to establish a more pragmatic approach to treatment [4, 18–61] in which non-surgical treatment plays an increasingly important role. Currently, there is a consensus that surgical decompression of the PA mass, most commonly via the transsphenoidal route, should be considered for any patient presenting with severe symptoms, which include severe visual field deficits and/or impaired consciousness [4, 62, 63]. However, whether surgery offers a superior long-term therapeutic benefit to conservative management remains unclear [4, 15–17, 62, 64]. Sibal et al. retrospectively reviewed 45 cases of PA and found that most patients experienced complete or near complete resolution of ophthalmologic deficits with no reported deaths following conservative management or surgery [16]. These findings were further supported in a recent multicenter, prospective study reported by Mamelak et al., in which investigators found no significant difference in outcomes in patients who underwent surgery compared to conservative management [4]. Many studies have documented little difference in hormonal outcomes between surgical and non-surgical patients, with only about 10% of patients recovering hormonal defects in either group (Table 3) [2, 4, 18, 19]. The resolution of cranial nerve palsies is comparable and often more favorable in conservatively managed patients at three to six-month follow-up [61]. 

**Table 3:** Comparison of initial endocrine deficiency compared to last follow-up in select studies

Author	Year	No. of cases	Cohort	Endocrine Deficiency (%)					Last follow-up (3 months or 1 year)			
				Corticotropic	Thyrotropic	Gonadotropic	Somatotropic	Hyponatremia	Corticotropic	Thyrotropic	Gonadotropic	Somatotropic
Grzywotz et al. [2]	2017	60	All	55	42.6	68.9	32	21.4	56.5	56.8	58.7	53.3
Marx et al. [18]	2021	19	Surgery	53.8	50	72.7	30.8	NR	64.7	70.6	80	60
		27	Conservative	57.1	54.2	66.7	8.7	NR	28.6	54.5	44.4	31.3
Shepard et al. [19]	2021	64	All	21.9	18.8	25	9.4	3.1	NR	NR	NR	NR
Mamelak et al. [4].	2024	67	Surgery	67	52	52	21	7.6	11.6*	8.9*	6.9*	NR
		30	Conservative	63	43	53	14	20	10.5*	8.3*	6.3*	NR

*Indicates % of patients who recovered from their endocrine deficiency at initial presentation. NR, not reported

In the largest observational study performed to date, symptoms, co-morbidities, demographics, and hormonal defects were almost identical between surgical and medical management cohorts, except that surgical patients had slightly larger tumors, but this difference was not significant [4]. The only statistically significant difference between these groups was the rate of bitemporal hemianopsia. Hospital stays were similar, though surgical patients had more complications. Patients with severe visual field defects and greater optic nerve compression were more likely to undergo surgery. At 3 to 6 months post-apoplexy, visual field, hormonal, oculomotor, and quality of life metrics were similar in both groups. The study did not show that surgery led to better visual outcomes, only that these criteria were commonly used to recommend surgery. This lack of outcome difference does not necessarily mean surgery is preferred for visual field defects, just that it was more often chosen.

**Fig. 1:**
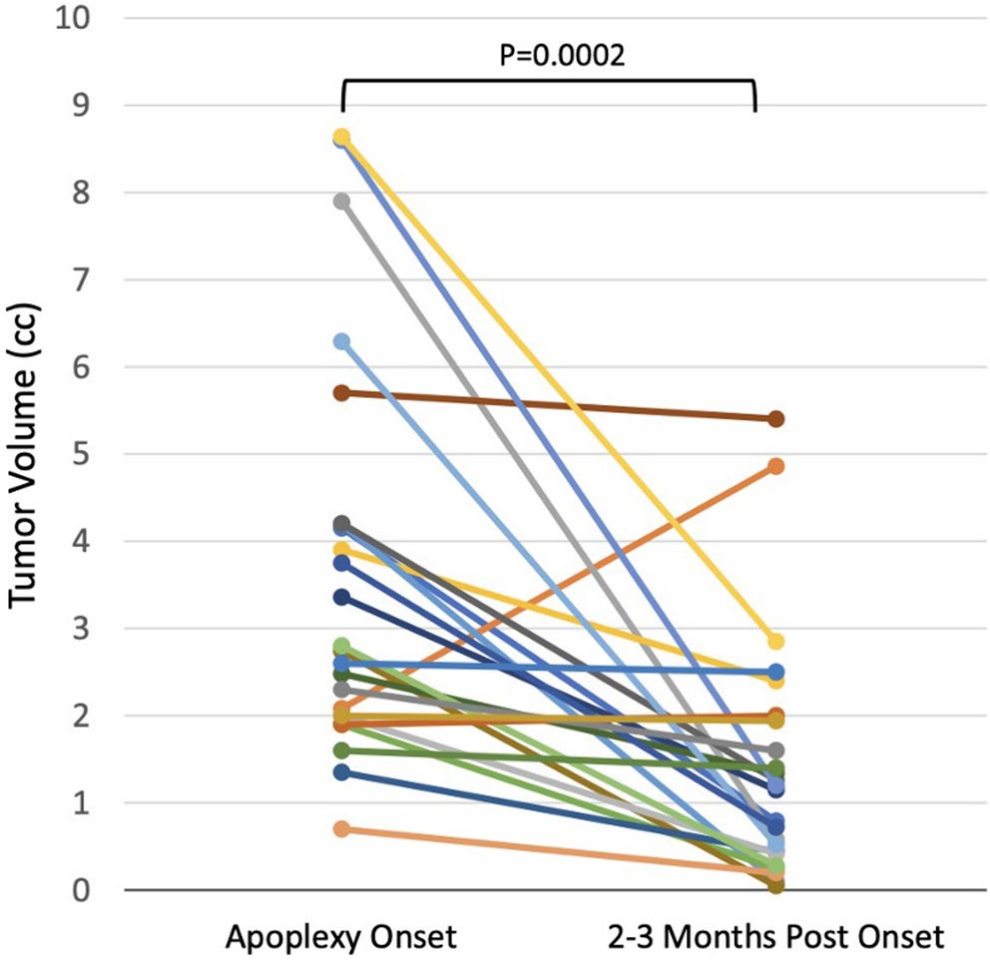
Change in Volume of Apoplectic Tissue in Medically Managed Patients by 2 to 3 Months After Symptom Onset. Volumes were measured using the (A × B × C)/2 approximation method. Each line represents one patient (*n* = 24). There was a significant 61% median reduction in volume over time (*P* = .0002), with only one patient showing volume enlargement. *Reproduced with permission from*: Mamelak, A. et al. (2023). A Prospective, Multicenter, Observational Study of Surgical vs. Nonsurgical Management for Pituitary Apoplexy. *Journal of Clinical Endocrinology & Metabolism, 109*(2), e711–e725. 10.1210/clinem/dgad541

An important but often overlooked aspect of PA is that the resulting mass effect is largely due to blood and edematous, necrotic tissue, which regress over time. Prior reports have noted regression in 70–95% of patients 3 months post apoplexy (**see** Fig. 1) [4, 19, 61]. Thus, symptom resolution due to diminished mass effect may be noted in most patients with PA, regardless of whether they undergo surgery. Whether these studies, often performed at centers with expert pituitary teams, represent general outcomes remains to be seen, particularly since growing evidence suggests that the degree of an institution’s experience in pituitary surgery is associated with overall outcomes [4, 65–67]. Despite the uncertainty in current literature, immediate decompression is generally safe and commonly used as the primary treatment modality in cases of severe visual deficits [68]. 

### Surgical timing

The optimal timing of surgical intervention when indicated is also not well defined. Patients presenting with severe cognitive impairment and visual deficits such as blindness should be promptly identified as urgent surgical candidates and likely undergo rapid decompression. However, such cases are relatively infrequent. Jho et al. found that patients with PA associated with more severe symptoms were more likely to undergo earlier surgery, similar to other reported studies [18, 62]. Establishing guidelines for intervention becomes more nuanced when addressing patients whose symptoms are stable or are showing signs of improvement. The challenge stems from the lack of evidence-based criteria guiding clinical decision-making in these scenarios.

The existing literature offers varied conclusions on the influence of early surgical intervention, and a consistent definition of “early surgery” is lacking. Woo et al. found that patients who underwent transsphenoidal surgery within three days of symptom onset experienced complete recovery of visual acuity, compared to 83% who had surgery later [69]. Furthermore, this study indicated that 66% of patients who underwent early surgery showed complete or partial recovery, compared to only 40% who received later interventions. These findings parallel those of Rutkowski et al., who ultimately found no differences in the rate of neurological and endocrine deficits following either early, defined as resection within three days of symptom onset, or delayed surgery. The authors also observed that earlier surgery was associated with greater gross total resection rates than delayed treatment (100% versus 44%, *p* = .003) despite similar size at the initial presentation [17]. Ultimately, no differences were found in the rates of neurological and endocrine deficits following either early or delayed surgery. Similarly, Bill et al. reported that patients who had surgery within seven days of symptom onset achieved complete recovery of visual functions [70]. Meanwhile, Randeva et al. reported that all patients who underwent surgery within eight days of symptom onset experienced complete recoveries [15]. 

A more comprehensive multi-center analysis compared outcomes by defining early surgery as either two, three, or four days post-symptom onset [4]. Investigators also considered 3-, 4-, and 5 days post-admission to the center where surgery was performed to exclude care delays. Their analysis indicated no advantage to any time-point cut-off for early surgery, with identical outcomes in the visual field, hormonal recovery, and oculomotor palsy regardless of the timing of surgical intervention. While patients who underwent surgery within these time frames exhibited more adverse visual field defects initially, their overall outcomes were comparable to those of patients who had surgery at 7 to 30 days post PA onset as well as those managed medically at the three-month evaluation mark.

### Management strategy based on best available data

In the acute clinical setting, once a presumptive diagnosis of PA has been made, pituitary axis hormones should be measured, and corticosteroids should be initiated immediately, even if lab results are unavailable, as this can always be discontinued if no longer indicated. Meticulous monitoring of vital signs and neurological status is crucial to detect any signs of clinical deterioration. Laboratory evaluations should include assessments of all pituitary axis hormones, electrolytes, renal function, liver function, coagulation parameters, and sodium levels. Monitoring urinary output and fluid balance is essential for detecting hyper or hyponatremia, which should be corrected rapidly. Depending on the institution’s protocols, adrenal insufficiency may be initially managed with a hydrocortisone bolus of 100 mg or equivalent, followed by a maintenance dose ranging from 10 to 40 mg in twice-daily doses. Thyroid-related deficiencies can be replaced slowly, and acute replacement of gonadotroph or somatotroph hormones is rarely indicated. If a patient is found to have a hormonally active prolactinoma, prompt initiation of medical therapy with dopamine agonists is likely indicated.

Once stabilized, an initial decision regarding acute neurosurgical intervention is appropriate. Patients with altered consciousness after corticosteroid replacement and correction of hyponatremia or with severe visual acuity diminishment should undergo surgery promptly, preferably within 48–72 h. Other patients can be observed for several days to monitor visual field stability or improvement, with surgery indicated only if deterioration occurs. This approach allows for transferring patients to specialized centers, improving outcomes. This approach also allows the transfer of patients to centers with greater expertise in pituitary surgery if needed, thereby reducing sup-optimal surgical outcomes. Discussion between the surgery team, endocrinologists, and the patient or family will determine the preferred treatment choice, with many patients opting for surgery, especially as this can often relieve headaches. In stable patients, the timing of surgery does not seem to impact outcomes greatly and can be done in an elective fashion within 2 to 10 days after onset. For patients with surgical risks, observation, and medical management may result in similar outcomes at 3 months due to regression of the apoplectic tissue and blood.

### Postoperative care and monitoring

Regardless of the management approach, all patients with PA require subsequent monitoring. MRI of the pituitary is important to evaluate for residual tumors, as these tumors can continue to grow. This is especially important in patients treated with medical management alone, as residual tumors have been reported to grow in 11–12% [20, 71]. Some of these patients may eventually undergo elective surgery to remove residual tumors even after the apoplectic event is resolved. Imaging studies should be conducted approximately three to six months after the apoplexy incident. Subsequently, annual MRI scans are recommended for a minimum of five years.

Hormone levels should be evaluated approximately four to eight weeks after an apoplexy event. Approximately 10 to 20% of patients achieve partial or complete recovery of pituitary function; however, about 80% will require ongoing hormonal supplementation or replacement therapy [2, 4, 18, 19]. GH deficiency is the most commonly observed endocrine deficiency in these patients, though it is infrequently replaced due to various factors, including clinical guidelines and patient-specific considerations. Long-term follow-up with endocrinology is recommended in most cases, especially if hormone supplementation or suppression (for functioning adenomas) is required. This protocol ensures comprehensive monitoring and management, maintaining appropriate neuroendocrine functions and quality of life.

## Conclusion

In the modern era, treatment strategies for PA have evolved considerably. Truly urgent cases of PA that require immediate and early (< 48 h) surgery for decompression are generally limited to patients with diminished levels of consciousness, severe visual defects, or progressively worsening exams. Fortunately, with prompt recognition of PA, these cases are relatively rare and, as such, are not commonly a true neurosurgical emergency. The reported association between COVID-19 infection or vaccination and PA should be kept in mind when patients present to medical attention, as many of the symptoms of PA can mimic those seen with acute COVID-19 infection. In most patients, correcting endocrine deficiencies, especially cortisol and hypo- or hypernatremia, will stabilize the patient. A decision for surgical intervention within the first 7–10 days after presentation can be made by the endocrine and neurosurgical services, as there is no clear evidence that earlier intervention results in better outcomes. Medical management without surgery is a highly effective strategy in many cases, partly due to the regression of apoplectic tissue and subsequent mass effect in most patients over time. Conducting a randomized trial to compare surgical and medical management appears warranted to define treatment strategies better, though designing and executing such a study would be challenging.

## Data Availability

No datasets were generated or analysed during the current study.
